# Comment on ‘Age of enlightenment: long-term effects of outdoor aesthetic lights on bats in churches’

**DOI:** 10.1098/rsos.171312

**Published:** 2017-11-15

**Authors:** Thierry Onkelinx

**Affiliations:** Research Institute for Nature and Forest, Kliniekstraat 25, 1070 Anderlecht, Belgium

**Keywords:** cultural heritage, historic buildings, biodiversity, light pollution, ordinal regression, mixed models

## Abstract

This comment reanalyses the data presented in Rydell (Rydell 2017 *R. Soc. open. sci.* **4**, 161077. (doi:10.1098/rsos.161077)) which were analysed using only very basic statistics like Fisher’s exact test and McNemar’s test. We demonstrate how the use of more advanced statistical methods can make better use of the available data, quantify the observed effects and strengthen the conclusions in Rydell (Rydell 2017 *R. Soc. open. sci.* **4**, 161077. (doi:10.1098/rsos.161077)). We have no intention to discredit the original authors. Their analyses were basic but correct.

## 1. Material and methods

The analysis was based on the original data [1] and run in R v. 3.4.1 [2]. The tidyverse [3] collection of packages was used for the data import, data wrangling and data visualization. ordinal [4] was used for the advanced analyses.

## 2. Results

### 2.1. The 2016 survey

The 2016 survey holds information on the presence of bats and the light status of the churches. Both variables can be represented as ordinal variables. The presence of the bats is recorded as no bats < used < colony. The light status is dark < partly lit < fully lit. After calculating a contingency table, we can apply Fisher’s exact test (*p*=0.0024). The interpretation of the test is limited: we reject the null hypothesis that the presence of bats and the light status are independent. It does not yield information on the effect size. To get around this, the 3×3 contingency table was recombined in several 2×2 tables. Each of those were subjected to Fisher’s exact test and the effect size was based on interpretation of the 2×2 tables. We found two such tests in the manuscript [1] and four in the dataset.

**Figure 1.**
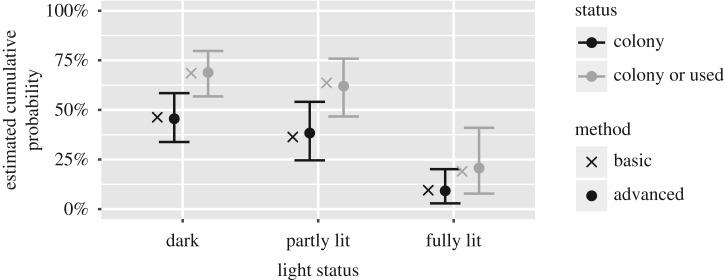
Estimated cumulative probability based on the 2016 survey using the basic and advanced method.

Another option is to analyse ordinal data with cumulative logit models [4]. The general form is given in equation (2.1). In this case, the presence of bats is the response variable and the light status is the covariate. We test for the overall effect of the light status with a likelihood ratio test (LRT) ($\chi_{2}^{2}=16.96$, *p*=0.00021). The predictions (figure 1) yield similar values as fig. 2 in [1].

2.1 $$\text{logit}(P(Y_{i}\leq j))=\theta_{j}-x_{i}^{T}\beta .$$

#### 2.1.1. Interpretation

Placing lights all around the exterior has strong negative effects on the presence of bats. The odds ratio (*OR*) for having no bats when fully lit compared with dark churches is *OR*: 9.40(3.04;35.94). The difference between dark and partly lit churches is unclear,^1^ *OR*: 1.38(0.62;3.08).

### 2.2. Comparison with 1980s survey

During the 2016 survey, all 60 churches of the 1980s survey have been revisited and 50 additional churches. The original authors take the repeated measures into account by using McNemar’s test [1]. This test works only on 2×2 tables.

A better alternative would be to use cumulative logit mixed models [4]. In this case, we use survey and light status as fixed effects and church as a random effect. This random effect takes the paired nature of the dataset into account. Note that the model separates the survey effect and the light status effect. The survey effect can model an overall decline in bats between surveys when the church remains dark. The interaction between survey and light status is not relevant as all churches were unlit in the 1980s survey. While McNemar’s test requires all data to be paired, the mixed model does not. Hence we can also use the data on the churches surveyed only in 2016.

**Figure 2.**
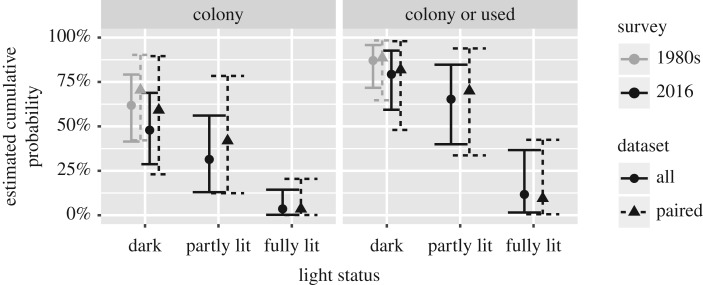
Estimated cumulative probability based on both surveys.

Let us see what the impact of using a larger dataset is. The LRT for the survey effect on the paired data is $\chi_{1}^{2}=0.4072$, *p*=0.52 and $\chi_{1}^{2}=1.343$, *p*=0.25 for the full data. Likewise we have $\chi_{2}^{2}=15.41$, *p*=0.00045 and $\chi_{2}^{2}=18.97$, *p*<0.0001 for the light status. The influence on the predictions is depicted in figure 2. The differences in predictions might be due to a possible bias in the 1980s survey, as the paired data yields higher estimates. Note that the confidence interval on the predictions are more narrow when we use the full data and that the estimates of the paired dataset are within the confidence intervals of the full data.

#### 2.2.1. Interpretation^2^

The difference in odds for finding no bats between both surveys is unclear, *OR*: 1.82(0.66;5.03). Likewise is the difference between dark and partly lit churches in 2016, *OR*: 2.09(0.55;7.90). The odds highly increase when changing from a dark to a fully lit church, *OR*: 40.61(4.16;396.85).

### 2.3. Effect of renovations

The renovation status is only available for churches from the 1980s survey, hence we can only use the paired dataset for this analysis. We added the renovation status as a fixed effect to the model. The interaction between lights status and renovation status would be relevant, but this gave a singular model.

#### 2.3.1. Interpretation

**Figure 3.**
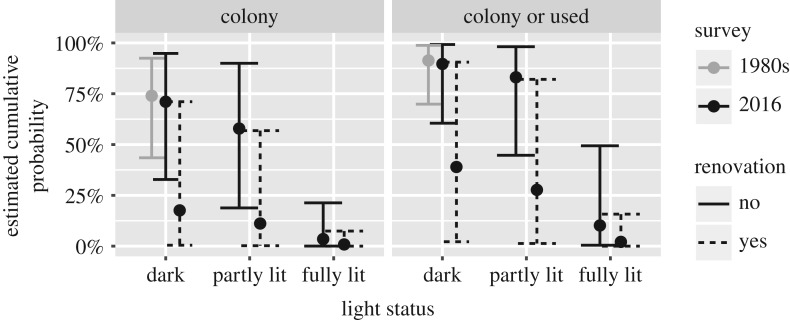
Estimated cumulative probability for the renovation model.

We find no evidence for an effect between surveys, *OR*: 1.12(0.26;4.87), $\mathrm{LTR}:\;\chi_{1}^{2}=0.02244,\;p=0.88$. The light status has a significant effect, $\mathrm{LTR}:\;\chi_{2}^{2}=16.9,\;p=0.00021$. We find an unclear effect of partly lit versus dark, *OR*: 1.93(0.23;16.17), but the odds for no bats between fully lit and dark increases, *OR*: 223.67(5.05;9897.81). Additionally, after renovation, the odds for no bats increase, *OR*: 24.85(1.00;616.55), $\mathrm{LTR}:\;\chi_{1}^{2}=5.419,\;p=0.02$. The predictions are given in figure 3. We could wonder which is worse: a fully lit, non-renovated church or a dark, renovated church. After calculating the odds ratio *OR*: 55.85(0.17;402.70), we must conclude that we cannot make a hard statement that one is worse than the other.

## 3. Discussion

We demonstrate how cumulative logit (mixed) models [4] are useful to analyse ordinal data and give more insight in the data.

When presenting the results to non-scientific readers like decision makers, we would use figure 3 because it summarizes the entire study in a single figure. The confidence intervals give a sense of the uncertainty associated with the results and reduce the need for stating *p*-values.

## Footnotes

1 Note that 1 is included in the confidence interval. *OR*=1 implies that both odds are equal, hence no effect.

2 Based on the full data.

## Data accessibility

The data and the code is available on https://github.com/ThierryO/rydell_etal_2017 (doi: 10.5281/zenodo.1002852).
